# Comparison of Sputum Treated with Power Ultrasound and Routine NALC-NaOH Methods for Mycobacterial Culture: A Prospective Study

**DOI:** 10.3390/jcm11164694

**Published:** 2022-08-11

**Authors:** Junling Wang, Yan Wang, Xiaojie Ling, Zhenjin Zhang, Yunfeng Deng, Peng Tian

**Affiliations:** Katharine Hsu International Research Center of Human Infectious Diseases, Shandong Key Laboratory of Infectious Respiratory Diseases, Shandong Public Health Clinical Center, Jinan 250101, China

**Keywords:** tuberculosis, decontamination methods, NaOH-N-acetyl cysteine, power ultrasound

## Abstract

Mycobacterial culture remains the gold standard for the diagnosis of active tuberculosis. However, an appropriate digestion and decontamination method is essential for the effective recovery of tubercle bacilli in culture. The study was designed to compare the efficacy of sputum treated with power ultrasound (PU) and routine NALC-NaOH methods for mycobacterial culture from clinically suspected cases of pulmonary tuberculosis. To evaluate the PU and routine NALC-NaOH methods, sputum specimens (*n* = 597) were studied (culturing on MGIT 960), and the performances were compared. Of the 597 samples, 89 (14.91%) sputum samples treated with the NaOH-NALC method were mycobacterial culture positive, including *Mycobacterium tuberculosis* (*M.TB*; *n* = 77, 12.90%) and nontuberculous mycobacteria (NTM; *n* = 12, 2.01%). One hundred and ten (18.43%) sputum samples treated with the PU method were culture positive, including *M.TB* (*n* = 87, 14.57%) and NTM (*n* = 23, 3.85%). The PU method detected 10 additional cases of M.TB and 11 additional cases of NTM when compared to the NALC-NaOH method. Statistical analysis showed that a significant difference was found in the culture-positive ratio of M.TB and NTM between the two method groups (*p* < 0.05). Compared with that of the NALC-NaOH method (8.04%), sputum treated with PU method (4.86%) had a significantly lower contamination rate (*p* < 0.05). In conclusion, our data indicate that, compared with the NALC-NaOH method, the PU method is a rapid and effective approach for mycobacterial culture when detecting active TB. However, its accurate mechanism has not been well addressed, and further investigation is still required.

## 1. Introduction

Tuberculosis (TB) remains a serious public health problem. It is estimated that TB infection affects one-third of the world’s total population. Early detection could decrease its prevalence and transmission [1]. As a gold standard, mycobacterial culture remains an efficient tool for the diagnosis of active TB. However, sputum usually contains a variety of microbiological agents that grow fast. Hence, before mycobacterial culture, digestion and decontamination are a challenge to be faced. During the past decades, various digestion and decontamination methods (DDMs) have been evaluated for sputum culture. However, not much progress has been made [2,3,4,5,6,7,8,9].

Ultrasound is known as a type of vibrational energy generated by sound waves, and can be classified into two major categories: high-frequency ultrasound, 2–20 MHz, and power ultrasound (PU, 20–100 kHz) [10]. PU has been widely used in biomedical research for several reasons; one is that PU can destroy bacteria. [11]. Further evidence suggested that different pathogens have variable sensitivity to PU [12,13]. This implies that the destructive activity of PU is pathogen specific [12,13,14,15,16]. This finding suggests the potential use of PU in sputum culture for *Mycobacterium tuberculosis* (*M.TB*). In this study, the PU method is introduced as a DDM, which consists of the PU technique and 1.7% sodium hydroxide (NaOH). Currently, the BACTEC MGIT 960 system, which is designed based on the liquid medium method, is recommended for culture by the World Health Organization (WHO) [1]. Therefore, we aimed to evaluate the role of the PU method for sputum mycobacterial culture (MGIT 960) and compare it with the routine N-acetyl-L-cysteine (NALC)-NaOH method.

## 2. Materials and Methods

### 2.1. Ethics

This study was carried out at the Katharine Hsu International Research Center of Human Infectious Diseases, Shandong Public Health Clinical Center. The protocol was approved by the Ethical Committee of Shandong Public Health Clinical Center (2019XKYYEC-27). 

### 2.2. Subjects

Between April and December 2019, new patients who were suspected of pulmonary TB (PTB) and produced sputum were recruited prospectively prior to administration of anti-TB therapy. 

The final diagnosis of PTB was based on composite reference standards (CRS), such as clinicoradiological findings, microbiological evidence (acid-fast bacilli (AFB) smear, polymerase chain reaction (PCR), and culture), and histopathological evidence from lung biopsies. Diagnostic yield, such as sensitivity was calculated against CRS. In addition, the contamination rate of each method was also recorded and analyzed.

According to the guidelines “Tuberculosis Laboratory Test Protocol (2015)”, morning sputum was collected from each patient on two consecutive days. Subsequently, sputum specimens were mixed in a Falcon tube and homogenized by vortexing (3 min) with small glass beads, facilitating the equitable distribution of bacterial communities. Besides an AFB smear (Auramine O method), the homogenized sputum was used to evaluate recovery efficiency and decontamination rate (NaOH-NALC vs. PU methods). Mycobacterial culture was performed using an MGIT 960 (Becton Dickinson, Franklin Lakes, NJ, USA), and the mycobacterial strains were identified by matrix-assisted laser desorption/ionization time-of-flight mass spectrometry (MALDI-TOF MS; Bruker Daltonics, Bremen, Germany) [17] or 16S rRNA gene sequencing [18].

### 2.3. DDM Methods

- NaOH-NALC method

Briefly, a pooled sputum was mixed with an equal volume of NaOH-NALC solution (4% NaOH, 2.9% sodium citrate, and 0.5% NALC; final 1% NaOH) using a vortex mixer, and then the mixture was incubated at room temperature for 15 min. Subsequently, 40 mL of sterile 0.067 M phosphate buffer (pH 6.8) was added and then centrifuged at 3000× *g* for 15 min. The supernatant was discarded, and the sediment was inoculated into MGIT medium.

- PU method

A noncontact PU meter (Scientz 08-IIIc, Ningbo, Zhejiang, China.) was used as the ultrasonic treatment system in this study. The parameters of sonication were performed using the following parameters: ultrasonic frequency, 20 kHz; input power, 19 W; power density, 4.75 W/mL; power intensities, 6.05 W/cm^2^; and sonication time, 1 min. The power density (D, W/mL) of the ultrasound dissipated into the medium with volume V is given by D = P/V, where P is the input power. The power intensity (I, W/cm^2^) dissipated from a probe tip with radius r is given by I = P/(πr^2^).

Concretely, a pooled sputum was mixed with an equal volume of 1.7% NaOH (final 0.85% NaOH) in a 50 mL BD Falcon centrifuge tube. Subsequently, the centrifuge tube was fixed on a special tube rack and put into the noncontact PU meter, treated with the above parameters of sonication. The centrifuge tube was removed from the PU meter immediately after sonication and incubated at room temperature for 5 min. Then, 40 mL sterile 0.067 M phosphate buffer (pH 6.8) was added to the mixture and centrifuged at 3000× *g* for 15 min. Finally, the sediment was inoculated into MGIT medium.

### 2.4. Statistical Analysis

The statistical analysis was performed using SPSS, version 15.0 (IBM, Armonk, NY, USA). Categorical variables were described using frequencies (percentages) and compared using the chi-square test, while continuous variables were described using mean (standard deviation) and compared using Mann–Whitney U test. The difference in recovery efficacy (sensitivity) and contamination rate between the two method groups was compared using the McNemar test. The agreement between the NaOH-NALC and PU methods was calculated using kappa value. A *p* value less than 0.05 was considered significant.

## 3. Results

### 3.1. Baseline Characteristics

**Figure 1:**
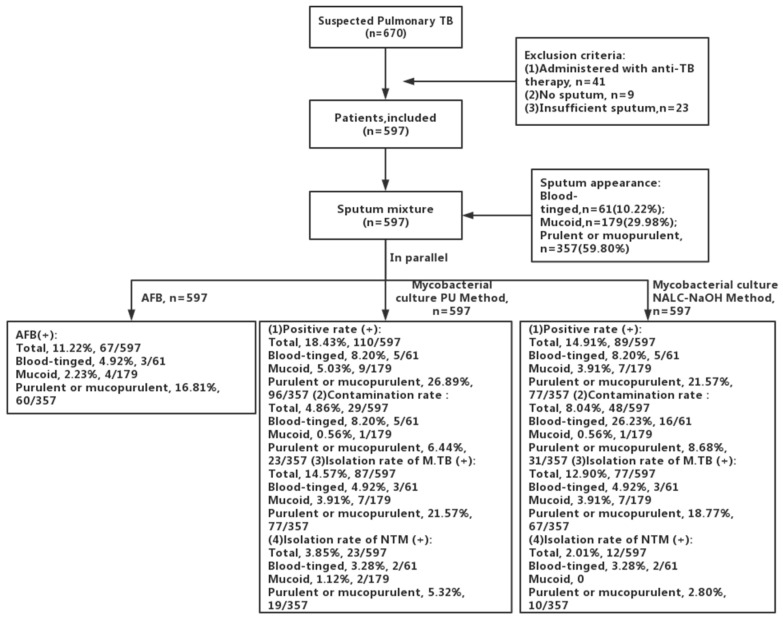
Patient selection.

A total of 597 patients (age, 49.57 ± 19.02 years; male, 66.16%, 395/597) were recruited in our study, and matched sputum was collected. Figure 1 shows the flow chart of patient selection. Of the 597 patients, 94 patients (15.75%) were diagnosed with pulmonary TB, and 503 (84.25%) patients had an alternative diagnosis. Among the 597 sputum specimens, 61 (10.22%) sputum samples were blood-tinged, 179 (29.98%) were mucoid, and 357 (59.80%) were purulent or mucopurulent. In addition, a total of 67 (11.22%) cases were AFB smear positive (Table 1). 

### 3.2. Recovery Efficacy (NaOH-NALC vs. PU Methods)

Of the 597 samples, 89 (14.91%) sputum samples treated with the NaOH-NALC method were mycobacterial culture positive, including *M.TB* (*n* = 77, 12.90%) and nontuberculous mycobacteria (NTM; *n* = 12, 2.01%). One hundred and ten (18.43%) sputum samples treated with PU method were culture positive, including *M.TB* (*n* = 87, 14.57%) and NTM (*n* = 23, 3.85%). The PU method detected 10 additional cases of *M.TB* and 11 additional cases of NTM when compared to the NALC-NaOH method. Statistical analysis showed that a significant difference was found in the culture-positive ratio of *M.TB* and NTM between the two method groups (*p* < 0.05). The agreement between the NaOH-NALC and PU methods obtained for *M.TB* was fairly good (kappa = 0.929, *p* < 0.001). However, regarding the isolation of NTM, the agreement was medium between the two method groups (kappa = 0.677, *p* < 0.001) (Table 2). 

### 3.3. Contamination Rate (NaOH-NALC vs. PU Methods)

Of the 597 sputum samples, 48 (8.04%) were contaminated using NALC-NaOH method, and 29 (4.86%) were contaminated using the PU method (Table 2). A significant difference between them was observed (*p* < 0.05). 

Among the 61 blood-tinged sputum samples, a significant difference in contamination rate was observed between the two methods (NALC-NaOH, 26.23%; PU, 8.20%; *p* < 0.05). However, no significant difference was found in mucoid and purulent or mucopurulent sputum when comparing NALC-NaOH and PU methods (Table 3). 

## 4. Discussion

As a gold standard for active TB, the performance of mycobacterial culture remains unsatisfactory. In our study, we found that the PU method could improve the isolation rate of NTM and *M.TB* in sputum. In addition, compared with that of the routine NALC-NaOH method, sputum treated with the PU method had a lower contamination rate. This interesting finding may help to improve the current situation of mycobacterial culture.

Although the NALC-NaOH method is widely used for treating sputum, regrettably, the NALC-NaOH method still has several limitations. For example, 1% NaOH is not only known as a decontamination agent but is also toxic to mycobacteria as well [3]. In addition, NALC should be prepared every day, this disadvantage limits the usefulness of the NALC-NaOH method [8]. Recently, several new agents were evaluated for digestion and decontamination. Cetylpyridinium chloride (CPC) and sodium chloride (NaCl) methods were confirmed to have a higher detection rate of TB than that of the NALC-NaOH method [9]. The use of sulfuric acid as the decontamination agent, instead of NaOH, has equal performance in TB detection with the NALC-NaOH method [6]. In general, although various DDMs have been developed to treat sputum, not much progress has been made in the field. 

In this study, the PU technique was successfully introduced to treat sputum for mycobacterial culture. Our data suggested that, compared with that of the NALC-NaOH method, more *M.TB* isolates were detected from sputum treated with the PU method. Compared with the NALC-NaOH method, the PU method has several advantages. First, a lower concentration of NaOH (0.85%) is used for decontamination. Second, the time for decontamination reduces from 15 to 6 min. Third, due to the noninclusion of NALC (which must be prepared and used fresh), PU method is easier to perform. Fourth, time to positivity (TTD), which reflects the metabolic activity of *M.TB* in liquid medium and has a negative correlation with the activity of inoculated bacteria, showed a relatively low level in the group using the PU method (data not published) [19]. This finding supports the position that the PU method is superior to the NALC-NaOH method in terms of preservation of cell activity. However, comparing the cost of the two methods, the PU method required an additional investment of about USD 5770 for the purchase of the PU meter. The approximate cost of culture per test (excluding labor and instrumentation) was found to be USD 8.6519 and 8.6597 for the PU method and the NALC-NaOH method, respectively.

Contamination is a concern for mycobacterial culture and remains a serious challenge for timely diagnosis. Although the PU method has a lower concentration of NaOH and shorter decontamination time, a better performance is observed in the contamination rate when compared with that of the NALC-NaOH method. Our data suggested that compared with that of the NALC-NaOH method (8.04%), sputum treated with PU method (4.86%) has a significantly lower contamination rate. Recently, the contamination rate of sputum treated with NALC and NaOH (1% to 2%) was estimated at 1.8%–13.4% [20,21,22]. Previously, an average contamination rate of 8.1% was reported in a multicenter study [23].

In addition, the PU method facilitated the detection of additional NTM cases. Facing the increasing incidence of NTM diseases, the PU method may have huge potential use. This advantage of the PU method may be explained by the difference in the response of NTM strains to various DDMs. However, further investigation is needed. This is because NTM is widely distributed in the environment, and contamination is unavoidable. Therefore, in this study, NTM culture was repeatedly performed in 23 patients, and data support our finding that the NTM isolation was not due to the contamination. 

Further analysis showed that sputum’s appearance could influence the culture results [24]. For example, blood-tinged sputum is easily contaminated when treated with the NALC-NaOH method, and the contamination rate was as high as 26.2% in our study. These data are consistent with previous studies [23]. However, the PU method could significantly reduce the rate of contamination. In addition, the PU method more efficiently decontaminated purulent or mucopurulent sputum than the NALC-NaOH method.

Although some interesting findings were obtained, the study has several limitations. First, the PU parameters were designed based on an experimental study (not published) of the *M.TB* strain (H37Ra) from artificial sputum. Compared with the *M.TB* and NTM isolated from the clinical samples in this study, these parameters may not be the optimal choice, which still needs to be further optimized. Second, to archive enough sputum, a pooled morning sputum was collected on consecutive days. This may increase the detection rate of *M.TB* and cannot reflect the real situation well. In addition, further large-scale and rigorous studies are still required to validate our findings.

In conclusion, our data indicate that, compared with the NALC-NaOH method, the PU method is a rapid and effective approach for mycobacterial culture when detecting active TB. However, its accurate mechanism has not been well addressed, and further investigation is still required.

## Figures and Tables

**Table 1:** Baseline characteristics of included patients.

Variables	Total (*n*)	Pulmonary TB * (*n*)
Number	597	94
Age, years	49.57 ± 19.02	50.28 ± 20.52
Male, %	66.16	60.64
HIV * status (+)	27	16
Extrapulmonary		
Pleural	34	5
Lymph node	11	1
Bone joint	7	
Sputum		
Blood-tinged	61	3
Mucoid	179	7
Purulent or mucopurulent	357	77
Microbiological evidence		
AFB * smear	597	50
PCR *	501	89
Culture	597	77
Underlying diseases		
Diabetes mellitus	32	5
Heart disease	43	11

* TB, tuberculosis; HIV, human immunodeficiency virus; AFB, acid-fast bacilli; PCR, polymerase chain reaction.

**Table 2:** Evaluation of AFB smear and culture results of suspected pulmonary TB patients using NaOH-NALC and PU methods of DDM.

		Total	Pulmonary TB (*n*)	Sensitivity ** (%)			Contamination Rate (%)
				Mycobacteria ***	NTM *	*M.TB*	
Culture	NaOH-NALC method	89	77	76.07 (67.13–83.26)	52.17 (31.08–72.58)	81.91 (72.34–88.82)	8.04
	PU * method	110	87	94.02 (87.62–97.35)	100 (82.19–100)	92.55 (84.75–96.70)	4.86
AFB	NaOH-NALC method	59	50	50.43 (41.09–59.74)	39.13 (20.47–61.22)	53.19 (42.66–63.46)	
	PU method	66	50	56.41 (46.94–65.45)	69.57 (46.99–85.94)	53.19 (42.66–63.46)	

* PU, power ultrasound; NTM, nontuberculous mycobacteria. ** Sensitivity (%) was calculated against a composite reference standard; *** including NTM and *M.TB*.

**Table 3:** Comparison of culture results for NaOH-NALC and PU methods based on sputum appearance.

		Total	Pulmonary TB (*n*)	Sensitivity * (%)			Contamination Rate (%)
				Mycobacteria **	NTM	*M.TB*	
NaOH-NALC method	Blood-tinged	5	3	83.33 (36.48–99.12)	100 (19.79–100)	75.00 (21.94–98.68)	26.23
	Mucoid	7	7	70.00 (35.36–91.91)	0 (0–80.21)	87.50 (46.68–99.34)	0.56
	Purulent or mucopurulent	77	67	76.24 (66.54–83.90)	52.63 (29.50–74.79)	81.71 (71.31–89.07)	8.68
	Total	89	77	76.07 (67.13–83.26)	52.17 (31.08–72.58)	81.91 (72.34–88.82)	8.04
PU method	Blood-tinged	5	3	83.33 (36.48–99.12)	100 (19.79–100)	75.00 (21.94–98.68)	8.20
	Mucoid	9	7	90.00 (54.12–99.48)	100 (19.79–100)	87.50 (46.68–99.34)	0.56
	Purulent or mucopurulent	96	77	95.05 (88.28–98.16)	100 (79.08–100)	93.90 (85.72–97.73)	6.44
	Total	110	87	94.02 (87.62–97.35)	100 (82.19–100)	92.55 (84.75–96.70)	4.86

* Sensitivity (%) was calculated against a composite reference standard; ** including NTM and *M.TB*.

## Data Availability

Not applicable.
